# Allelopathic Potential of Newly Emerged Invasive Plant *Cirsium vulgare* (Asteraceae) in Yunnan Province of China

**DOI:** 10.3390/plants15030513

**Published:** 2026-02-06

**Authors:** Fengping Zheng, Che Zhan, Kexin Yang, Qiurui Li, Zhijie Wang, Gaofeng Xu, David Roy Clements, Bin Yao, Guimei Jin, Shaosong Yang, Fudou Zhang, Michael Denny Day, Shicai Shen

**Affiliations:** 1 Key Laboratory of Prevention and Control of Biological Invasions, Agricultural Environment and Resource Research Institute, Yunnan Academy of Agricultural Sciences, Ministry of Agriculture and Rural Affairs of China, Kunming 650205, China; zfping668@163.com (F.Z.); zhanche@mail.kib.ac.cn (C.Z.); 18869574002@163.com (Q.L.); w1330549167@163.com (Z.W.); xugaofeng1059@163.com (G.X.); yaobin1019@163.com (B.Y.); jgmbly2006@126.com (G.J.); yshaos@163.com (S.Y.); fdzh@vip.sina.com (F.Z.); 2 Key Laboratory of Green Prevention and Control of Agricultural Transboundary Pests of Yunnan Province, Agricultural Environment and Resource Research Institute, Yunnan Academy of Agricultural Sciences, Kunming 650205, China; 3 Yunnan Lancang-Mekong Agricultural Bio-Security International Science and Technology Cooperation Joint Research Center, Agricultural Environment and Resource Research Institute, Yunnan Academy of Agricultural Sciences, Kunming 650205, China; 4 School of Life Sciences, Jiangwan Campus, Fudan University, 2005 Songhu Road, Shanghai 200437, China; kxyang25@m.fudan.edu.cn; 5 School of Agriculture, Yunnan University, Kunming 650504, China; 6 Department of Biology, Trinity Western University, Langley, BC V2Y 1Y1, Canada; clements@twu.ca; 7 Queensland Department of Primary Industries, P.O. Box 267, Brisbane, QLD 4001, Australia; michael.day@dpi.qld.gov.au

**Keywords:** invasive species, allelopathic potential, phenolic acid, flavonoid, allelopathic index

## Abstract

*Cirsium vulgare* (Asteraceae) is a newly emerged invasive species in Yunnan Province, China, and its phytotoxic potential has not yet been studied. This study was conducted to explore potential allelopathic effects of *C. vulgare* and to identify its flavonoid and phenolic acid compounds. Four aqueous extracts (roots, stems, leaves, and flower/fruit heads) of *C. vulgare* exhibited high inhibitory activity against the germination and seedling growth of *Bidens pilosa* and *Digitaria sanguinalis*. The inhibition rates of germination rate, germination index, root length, shoot length, and biomass of both species increased significantly with increasing concentrations, with *B. pilosa* being more inhibited than *D. sanguinalis*. Extracts from leaves and flower/fruit heads yielded the strongest inhibition, followed by stem extracts, with the lowest impact from root extracts. Flavonoids (65.41%) and phenolic acids (23.1%) collectively comprised 88.51% of all identified compounds. Thirty-eight flavonoid compounds and thirty phenolic acid compounds were selected for further analysis, representing 53.97% and 71.91% of the total content of flavonoids and phenolic acids, respectively. Many of the flavonoids and phenolic acids identified have been previously reported as known allelochemicals with possible allelopathic effects. This was the first study to show that the allelopathic potential of *C. vulgare* may aid its invasion and expansion.

## 1. Introduction

Invasive alien plant species are highly impactful and pose a major threat to global biodiversity loss [1]. They not only disrupt critical ecosystem services but also cause various negative impacts to socio-economic development [2,3,4]. Many invasive species dominate ecosystems owing to their rapid growth and strong physiological and ecological adaptability, and are frequently allelopathic [5,6,7,8]. A study of 524 invasive plant species found that over half (51.4%) the species produce allelochemicals, demonstrating the potential for allelopathy to significantly impact native species and crops worldwide [2]. Furthermore, allelopathy is commonly regarded as a main contributing factor in the success of these invasive exotics [6,9,10]. The novel weapon hypothesis [6] and allelopathic-driving hypothesis [8] demonstrate that invasive species may produce allelochemicals that can directly inhibit neighboring native plants or indirectly suppress native plants via disruption of beneficial below-ground microbial mutualisms, or altered soil resources. Therefore, studying the allelopathic potential of invasive plants is essential to understanding how they compete with crops and native species.

The biennial to short perennial herbaceous plant *Cirsium vulgare* (Savi) Ten. (Asteraceae) is native to Europe, western Asia, and North Africa [11,12]. *Cirsium vulgare* has expanded its range to cool-temperate, warm-temperate, and subtropical zones on every continent except Antarctica by thriving in human-altered habitats [13]. Consequently, it is regarded as one of the world’s most notorious invasive plants, being both highly impactful to the environment and geographically widespread [14,15,16]. This species thrives in various open and disturbed habitats, including arable field margins, roadsides, forest clearings, and pastures [12].

*Cirsium vulgare* was first recognized as a species of potential concern in Yunnan Province, southwestern China in 2022. A specimen was collected from Panlong District, Kunming City, and subsequently identified as *C. vulgare* through comprehensive literature review and expert opinion. It has been deposited in the Agricultural Environment and Resource Research Institute, Yunnan Academy of Agricultural Sciences, China. Further field investigations and monitoring across the province showed that *C. vulgare* is common in Kunming City, Yuxi City, Chuxiong City, and Qujing City, frequently invading farmland, wasteland, and managed public landscapes such as parks, roadsides, and green belts. iNaturalist records show that it also occurs in Guizhou Province. This species is characterized as an injurious weed due to its spiny stems and leaves that make it unpalatable to most stock, although seedlings or small rosettes may be eaten by sheep [12,14]. The robust taproot of *C. vulgare* allows its adaptability to diverse environmental conditions, particularly drought [12]. *Cirsium vulgare* can easily spread to surrounding areas by wind through numerous pappus hairs around seeds.

*Cirsium* species contain secondary metabolites such as flavonoids, phenolic acids, sterols, and aliphatic aldehydes [17,18,19,20], which are known to have various antioxidant and antibacterial activities [21,22,23]. Phenolic acids and flavonoids have been extensively studied for their allelopathic activity [24,25,26]. Phenolic allelochemicals mediate plant competition through the suppression of growth of other species, thereby influencing competitive interactions and thus affecting plant succession [27]. Previous studies have shown, using a range of extraction methods, that *C. vulgare* contains different phenolic acid and flavonoid compounds in various plant parts, and that these compounds have strong antioxidant activity [20,22,25]; however, no investigations into phytotoxic activity have been conducted.

This study hypothesized that *C. vulgare* may have strong competitiveness through its allelopathic potential. In order to explore the allelopathic mechanisms, the allelopathic potential of *C. vulgare* against two weed species, *Bidens pilosa* L. (Asteraceae) and *Digitaria sanguinalis* (L.) Scop. (Poaceae), which are widespread in China and commonly found in areas with *C. vulgare*, was investigated using aqueous extracts from the roots, stems, leaves, and flower/fruit heads of *C. vulgare*. Moreover, based on initial bioassay results and previous studies on phenolic acid and flavonoid compounds of *C. vulgare* [20,22,25], the overall phenolic acids and flavonoids from methanol extracts of *C. vulgare* leaves were explored by UPLC-MS analysis. Understanding the allelopathic mechanisms of the invasive plant *C. vulgare* is crucial for developing early detection and rapid response strategies for proactive management.

## 2. Results

### 2.1. Seed Germination and Seedling Growth

Seed germination and seedling growth of *B. pilosa* and *D. sanguinalis* varied significantly with plant part and concentration level of *C. vulgare* extracts, with a significant interaction effect (except for the germination index of *B. pilosa*) (*p* < 0.01) (Table 1 and Table 2). The germination rate and germination index of *B. pilosa* and *D. sanguinalis* were significantly inhibited by all four aqueous extracts of *C. vulgare*, with the highest inhibition rates by leaves and flower/fruit heads, followed by stems, and the lowest impact was seen from root extracts (Table 1). With increasing concentrations of the four aqueous extracts, inhibition was markedly increased, and the inhibitory rates of the germination index of the two test species were generally higher than those of germination rate. The inhibitory rates of germination rate and germination index of *B. pilosa* were generally higher than those of *D. sanguinalis* (Table 1).

The four aqueous extracts of *C. vulgare* resulted in varying effects on the root length, shoot length, and biomass of *B. pilosa* and *D. sanguinalis* (Table 2). The four aqueous extracts of *C. vulgare* exhibited powerful inhibitory effects on seedling growth of the two test species (Table 2). The inhibitory rates of the aqueous extracts on the root length, shoot length, and biomass of *B. pilosa* and *D. sanguinalis* significantly increased with higher extract concentrations, with *B. pilosa* experiencing greater inhibition than *D. sanguinalis*. Against *B. pilosa*, extracts from leaves and flower/fruit heads of *C. vulgare* generally had the strongest inhibition effect, followed by stems, with the lowest inhibition from root extract. However, against *D. sanguinalis,* extracts from leaves generally had the strongest inhibition effect on the root length, shoot length, and biomass (Table 2).

### 2.2. Allelopathic Response Index

The measured allelopathic response index and the synthetical allelopathic index of the aqueous extracts of *C. vulgare* on seed germination and seedling growth of *B. pilosa* and *D. sanguinalis* varied, depending on plant parts, concentrations, and species (Table 3 and Table 4). All measured allelopathic response indices of the aqueous extracts of *C. vulgare* on *B. pilosa* and *D. sanguinalis* were significantly lower than 0 and were significantly reduced with increasing concentration. Comparing the allelopathic response index for the two bioassay species, germination rate was inhibited minimally more than germination index, with shoot and root length and biomass the highest inhibited, and the inhibitory rates of *B. pilosa* were generally higher than those of *D. sanguinalis* (Table 3 and Table 4). Comparing the synthetical allelopathic index values across both *B. pilosa* and *D. sanguinalis*, the magnitude of the inhibition was greatest for leaf and flower/fruit head extracts generally, with somewhat less inhibition for stem extracts, and the least inhibition by root extracts (Table 3 and Table 4).

### 2.3. Identification of Flavonoid and Phenolic Acid Compounds

**Figure 1:**
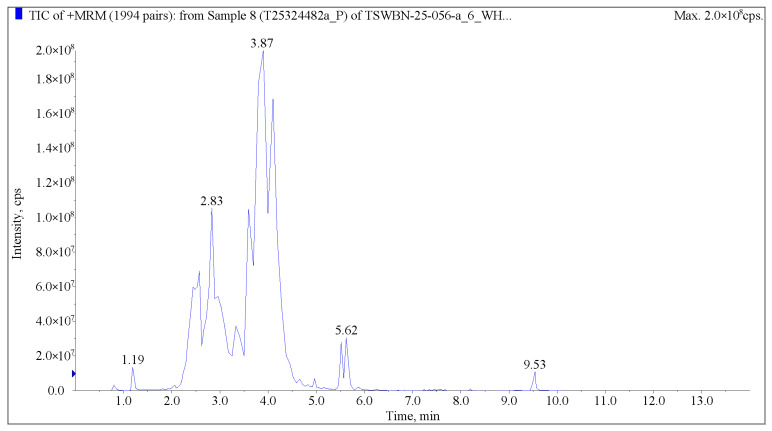
Total ions current chromatogram (by UPLC-MS) of some main phenolic acid and flavonoid compounds from methanol extracts of *Cirsium vulgare*.

**Figure 2:**
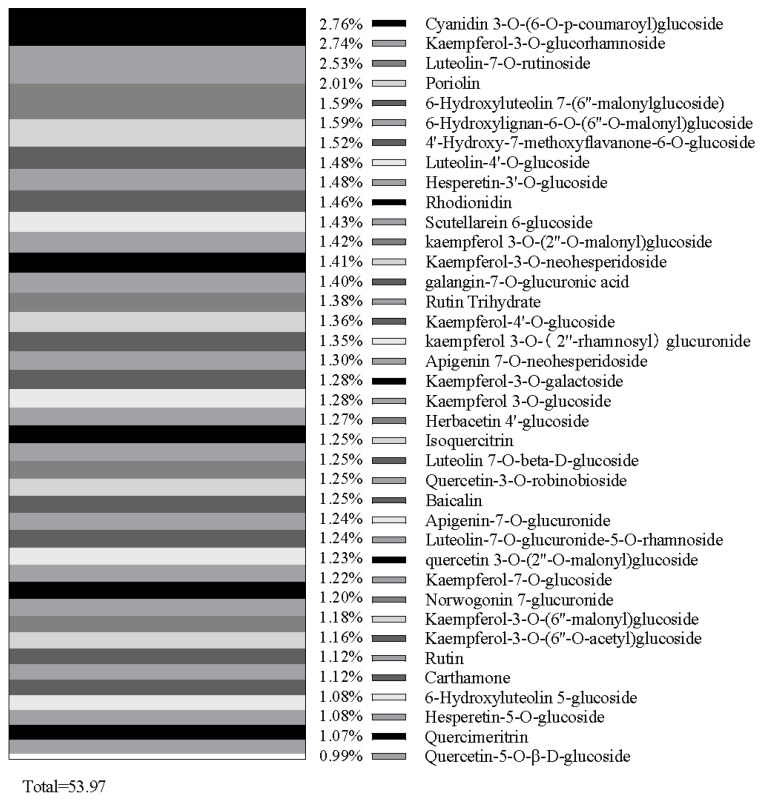
Main flavonoid compounds of methanol extracts of *Cirsium vulgare*.

**Figure 3:**
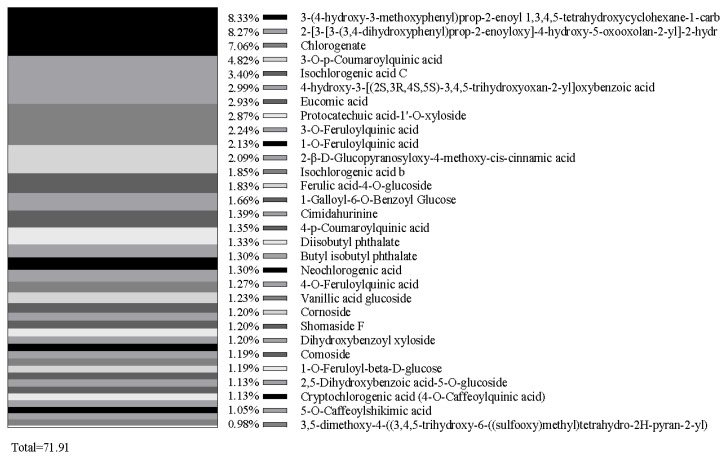
Main phenolic acid compounds of methanol extracts of *Cirsium vulgare*.

Flavonoids (65.41%) and phenolic acids (23.1%) collectively comprise 88.51% of all identified compounds resulting from the methanol extracts of *C. vulgare* leaves. Thirty major compounds, such as 3-(4-hydroxy-3-methoxyphenyl)prop-2-enoyl 1,3,4,5-tetrahydroxycyclohexane-1-carboxylate (2.17%), 2-[3-[3-(3,4-dihydroxyphenyl)prop-2-enoyloxy]-4-hydroxy-5-oxooxolan-2-yl]-2-hydroxyacetic acid (2.16%), cyanidin 3-O-(6-O-p-coumaroyl) glucoside (2.04%), kaempferol-3-O-glucorhamnoside (2.02%), luteolin-7-O-rutinoside (1.87%), and chlorogenate (1.84%), from flavonoids and phenolic acids, were selected (Figure 1 and Table 5). Moreover, thirty-eight flavonoid compounds and thirty phenolic acid compounds occupied by near and over 1.0% of content were selected, representing 53.97% and 71.91% of the total content of flavonoids and phenolic acids, respectively (Figure 2 and Figure 3). Many compounds of these flavonoids and phenolic acids are known allelochemicals produced by other plant species and their phytotoxic effects have been previously reported.

## 3. Discussion

The current study demonstrated that aqueous extracts of *C. vulgare* exhibited phytotoxic activity, significantly inhibiting seed germination and seedling growth of *B. pilosa* and *D. sanguinalis*. Increasing numbers of studies have indicated that allelopathy is one of the main drivers for biological invasions and many invasive plant species can inhibit growth of neighboring plants via allelopathy [6,10,28,29]. However, with *C. vulgare* being a newly reported invasive plant species for Yunnan Province, China, its potential allelochemicals and its allelopathic effects on neighboring plants were unknown prior to the present study.

This study showed that the phytotoxic effects of aqueous extracts of *C. vulgare* increased with increasing extract concentration, which was consistent with many other research studies [10,30,31]. There were obvious differences in species-specific growth inhibitory activities from the various plant extracts. Inhibitory rates were generally higher for extracts from flower/fruit heads and leaves compared to stems, with the lowest rates of inhibition observed for roots, suggesting that the presence of phytotoxic substances may be richer in leaves and flower/fruit heads than in roots. Other studies have also shown that above-ground parts have greater allelopathic effects than other parts [10,32,33]. Fernandez et al. [33] reported that the leaves of *Pinus halepensis* Mill. (Pinaceae) contained higher levels and a greater variety of allelochemical compounds compared to its roots. Shen et al. [10] found that the invasive plant *Acmella radicans* (Jacq.) R.K.Jansen (Asteraceae) had allelopathic potential on *Brassica rapa* L. (Brassicaceae) and *Chrysanthemum coronarium* L. (Asteraceae) (now known as *Glebionis coronaria* (L.) Cass. ex Spach), with the overall inhibition rates ranked in the order flower/fruit head > leaf > stem > root.

Flavonoids (65.41%) and phenolic acids (23.1%) accounted for 88.51% of all identified compounds in the methanol extracts of *C. vulgare* in this study. Thirty-eight major flavonoid compounds and thirty major phenolic acid compounds were selected, representing 53.97% and 71.91% of the total content of flavonoids and phenolic acids, respectively. Many compounds of these flavonoids and phenolic acids such as ferulic acid [26], vanillic acid [30], proline [34], rutin [35], apigenin [36], luteolin [37], quercetin [38], kaempferol [39], isochlorogenic acid [40], and protocatechuic acid [41] have been previously reported to be known allelochemicals and have possible allelopathic effects. However, the phytotoxic potential of some compounds identified from the methanol extracts of *C. vulgare* still remains unclear and requires further investigation.

Allelopathic compounds can be released at any time during the life cycle of plants under natural conditions, but the allelopathic potential of different plant parts under different extraction methods varies greatly [42,43]. Some previous studies have shown that *C. vulgare* contains phenolic acid and flavonoid compounds and has strong antioxidant activity, but this depends on plant part and extraction method. Kozyra and Głowniak [20] found that the methanol extracts from dried flowering herbs of *C. vulgare* have richer phenolic acids and higher content compared to the water extracts from the leaves [22]. The highest total flavonoid content from whole plant parts of *C. vulgare* was found using methanol extracts, followed by ethyl acetate and diethyl ether extracts, while no flavonoids were found from hexane extracts [25]. Moreover, the highest yield of phenolic compounds from dried leaves of *C. vulgare* was produced by heat-reflux extraction for 90 min using 50% ethanol as a solvent [44]. Different phenolic compounds were obtained from various petrol, chloroform, and methanolic extracts of *C. vulgare* dried flower heads [45]. The methanolic and aqueous extracts and fractions of dried inflorescences and leaves of *C. vulgare* generally showed antioxidant and antimicrobial activity, which depends on phenolic compounds [19,21,22,45]. The current study found that there are abundant phenolic acid and flavonoid compounds in fresh leaves of *C. vulgare* for its phytotoxic effects, although they may differ between various plant parts and with different extraction methods.

Currently, *C. vulgare* is seen as an emerging serious invasive plant and most populations are distributed in Central Yunnan Province, China. In a separate modeling study, MaxEnt analysis showed that the potential suitable area of *C. vulgare* in China is predominantly concentrated in the northwest, southwest, and central regions of the mainland, as well as Taiwan. From field observations, this species is well-adapted to urban environments and is commonly found in green areas such as parks, flower beds, wastelands, and roadsides across cities and suburbs. *Cirsium vulgare* usually exhibits a competitive advantage in plant communities due to its high plant height, broad leaves, deep root system, and injurious spines. The present study showed that *C. vulgare* has strong allelopathic potential against seed germination and seedling growth of two common weeds in Yunnan Province through releasing phenolic acid and flavonoid compounds. These were primarily released in leaf and flower/fruit head leachates. The strong allelopathic potential of *C. vulgare* against *B. pilosa* and *D. sanguinalis* suggests that its management strategies must account for chemical interference, not merely resource competition. Therefore, in order to reduce both seed dispersal and allelochemicals inputs into the soil, early removal of above-ground biomass and plant residues of *C. vulgare* should be considered. In addition, applications of the phytotoxicity of *C. vulgare* could be explored such as in suppressing neighboring weed species or developing novel, plant-derived weed management tools.

## 4. Materials and Methods

### 4.1. Plant Materials Collection

Cirsium vulgare is biennial, and occasionally behaves as an annual under favorable conditions. It is widely distributed in Kunming City of Yunnan Province of China. Whole plants of *C. vulgare* were collected during the flowering and fruiting period in green belts of Yunnan Academy of Agricultural Sciences, China in June 2024. After harvesting, fresh plant samples were divided into four groups (roots, stems, leaves, and flower/fruit heads) and then cut into pieces (approximately 1–2 mm) for aqueous extraction and bioassay.

### 4.2. Test Plant Species, Aqueous Extract Preparation, and Bioassay

Two test species, *B. pilosa* and *D. sanguinalis* were used for biological assay. *Bidens pilosa* and *D. sanguinalis* are noxious weeds and commonly occur in temperate, subtropical, and tropical regions in Yunnan Province.

The extracts of roots, stems, leaves, and flower/fruit heads of *C. vulgare* were all prepared using the same method: 50 g of fresh tissue segments was immersed in 500 mL of distilled water (10% *w*/*v*) at room temperature (20 ± 5 °C). The extracts were stored in a refrigerator for 48 h before filtering. Filtration was performed by first passing the extracts through two layers of cheesecloth and then through two layers of filter paper. The filtered aqueous extracts had an initial concentration of 0.1 g·mL^−1^. For subsequent bioassays, the extracts were then diluted with distilled water to four concentrations (0.0125, 0.025, 0.05, and 0.1 g·mL^−1^), as established in pre-experimental trials. The extract concentrations were relative low compared to some similar studies in which aqueous extracts from dried or fresh materials were prepared [10,32,33]. All solutions were stored at 4 °C until use.

To evaluate the allelopathic effects of *C. vulgare * on the two test species, the four extract concentrations and a control (distilled water, CK) were tested, each with four replicates. Twenty surface-sterilized seeds of each test species were evenly placed in separate Petri dishes (9 cm diameter) containing two layers of filter paper, and 5 mL of extract or distilled water (control) were added in each treatment. The bioassay procedures for assessing the effects of the different concentrations on the germination of seeds and biomass of seedlings of the two plant species were identical to those described by Shen et al. [46]. Germination counts were made daily, while shoot height, root length, and seedling fresh biomass were determined after the 7-day experimental period.

### 4.3. Identification of Flavonoid and Phenolic Compounds

Based on initial bioassay analysis and previous studies on phenolic acid and flavonoid compounds of *C. vulgare* [20,22,25], leaves were selected for further study using the methanol extract method. The young fresh leaves of *C. vulgare* were collected and placed in a lyophilizer (Scientz-100F, Scientz, Ningbo, China) for 63 h, then ground (30 Hz, 1.5 min) to powder form using a grinder (MM 400, Retsch, Haan, Germany). Then, 30 mg of sample powder was weighed using an electronic balance (MS105DΜ) and 1500 μL of −20 °C pre-cooled 70% methanol aqueous internal standard extract (less than 30 mg added at the rate of 1500 μL extractant per 30 mg sample) was added. The samples were placed in a Vortex once every 30 min for 30 s, for a total of 6 times. After centrifugation (rotation speed 12,000 rpm, 3 min), the supernatant was aspirated, and the sample was filtered through a microporous membrane (0.22 μm pore size) and stored in the injection vial for UPLC-MS/MS analysis.

### 4.4. UPLC-MS Analysis

The sample extracts were analyzed using an ultraperformance liquid chromatography−electrospray ionization tandem mass spectrometry (UPLC-ESI-MS/MS). The analytical conditions of UPLC were as follows: Column, Agilent SB-C18 (1.8 µm, 2.1 mm × 100 mm; Agilent, Santa Clara, CA, USA). The mobile phase consisted of solvent A (pure water with 0.1% formic acid) and solvent B (acetonitrile with 0.1% formic acid). Sample measurements were performed with a gradient program that employed starting conditions of 95% A, 5% B. Within 9 min, a linear gradient to 5% A, 95% B was programmed, and a composition of 5% A, 95% B was kept for 1 min. Subsequently, a composition of 95% A, 5.0% B was adjusted within 1.1 min and kept for 2.9 min. The flow velocity was set as 0.35 mL per minute, the column oven was set to 40 °C, and the injection volume was 2 μL. The effluent was alternatively connected to an ESI−triple quadrupole−linear ion trap (QTRAP)-MS.

The MS conditions were as follows: the source operation parameters of electrospray ionization (ESI) included source temperature 500 °C, ion spray voltage (IS) 5500 V (positive ion mode)/−4500 V (negative ion mode), ion source gas I (GSI) (50 psi), gas II (GSII) (60 psi), curtain gas (CUR) (25 psi), and high collision-activated dissociation (CAD). The triple quadrupole (QQQ) scans were acquired as multiple reaction monitoring (MRM) experiments with collision gas (nitrogen) set to medium. The declustering potential (DP) and collision energy (CE) for individual MRM transitions were done with further DP and CE optimization. A specific set of MRM transitions was monitored for each period according to the metabolites eluted within this period.

Metabolite identification was performed using secondary mass spectral data. After obtaining the mass spectrometry data of metabolites from different samples, peak area integration was conducted for all substance chromatographic peaks, and integration correction was applied for the same metabolite detected across different samples [47].

### 4.5. Statistical Analysis

Data were analyzed using IBM SPSS software version 23.0 (Armonk, New York, NY, USA). The inhibitory rates (IRs) of aqueous extracts of *C. vulgare* for germination rate, germination index, plant height, and biomass were calculated using the formula (C − T)/C × 100%, where C is the mean value of control, and T is the mean value of each extract treatment (IR > 0 indicates inhibition, IR < 0 indicates promotion, and the magnitude of IR values reflects the intensity of the allelopathic effect) [48]. The allelopathic response index (RI) was calculated for aqueous extracts from *B. pilosa* and *D. sanguinalis.* The formula for RI is: when T ≥ C, RI = 1 − C/T; when T < C, RI = T/C − 1, where C is the control value and T is the treatment value; R > 0 indicates promotion, IR < 0 indicates inhibition, and the magnitude of IR values reflects the intensity of the allelopathic effect [49]. A synthetical allelopathic index was then derived by averaging the RI values obtained for germination rate, germination index, root length, shoot length, and biomass. The result data were analyzed using two-way ANOVA to evaluate plant part and concentration levels, with their interaction (ns, *, and ** indicate *p* > 0.05, *p* ≤ 0.05, and *p* ≤ 0.01, respectively, for *F* value), and one-way ANOVA to evaluate inhibitory rate and allelopathic response index (5% significance level). Duncan’s multiple range tests were conducted to compare treatment differences (*p* < 0.05) for significant differences detected by ANOVA.

## 5. Conclusions

This study showed that extracts of *C. vulgare* inhibited the growth of *B. pilosa* and *D. sanguinalis*. The inhibitory rates of the aqueous extracts of *C. vulgare* on seed germination and seedling growth of *B. pilosa* were generally higher than the rates for *D. sanguinalis*. The germination rate, germination index, root length, shoot length, and biomass of the two test species significantly declined as the concentration of *C. vulgare* extracts increased. Comparing the allelopathic response indices derived from the four aqueous extracts of *C. vulgare* on *B. pilosa* and *D. sanguinalis*, flower/fruit heads and leaves yielded the strongest inhibition, followed by stems, with the lowest impact from root extracts. Thirty-eight major flavonoid and thirty major phenolic acids were identified from the methanol extracts of *C. vulgare*, with many compounds, including ferulic acid, vanillic acid, proline, rutin, apigenin, luteolin, quercetin, kaempferol, isochlorogenic acid, and protocatechuic acid having been reported to have possible allelopathic effects and considered as potential allelochemicals in previous research. However, the phytotoxic potential of some compounds from the methanol extracts of *C. vulgare* is still not clear and needs to be further tested, although phenolic compounds have been considered as potential allelochemicals. Therefore, complementary field-based studies and further studies on the allelochemicals and allelopathic mechanisms of *C. vulgare* under various conditions would provide valuable additional information on how the allelopathic potential of *C. vulgare* facilitates its invasiveness.

## Figures and Tables

**Table 1:** Inhibitory rate (%) of *Cirsium vulgare* extracts on seed germination of *Bidens pilosa* and *Digitaria sanguinalis*.

Plant Part	Concentration (g·mL^−1^)	*Bidens pilosa*		*Digitaria sanguinalis*	
		Germination Rate	Germination Index	Germination Rate	Germination Index
Root	0.1	26.58 ± 3.28 a	46.01 ± 3.29 a	18.57 ± 4.97 a	20.66 ± 2.06 a
	0.05	21.24 ± 3.47 a	31.32 ± 3.58 b	14.55 ± 6.49 a	17.02 ± 1.83 a
	0.025	15.89 ± 3.67 ab	16.17 ± 4.18 c	13.30 ± 2.88 a	9.76 ± 2.67 b
	0.0125	9.02 ± 8.49 b	9.48 ± 5.50 c	11.92 ± 4.91 a	3.65 ± 3.91 c
Stem	0.1	43.93 ± 3.59 a	60.74 ± 1.30 a	21.27 ± 3.95 a	32.30 ± 1.94 a
	0.05	33.24 ± 3.91 ab	48.63 ± 8.02 b	19.96 ± 4.82 a	26.27 ± 2.85 b
	0.025	22.55 ± 4.28 bc	31.57 ± 2.76 c	18.57 ± 4.97 a	20.89 ± 2.72 c
	0.0125	14.36 ± 8.23 d	19.83 ± 4.22 d	16.01 ± 0.44 a	16.11 ± 1.62 c
Leaf	0.1	53.30 ± 2.54 a	69.48 ± 0.52 a	53.29 ± 3.31 a	62.02 ± 2.03 a
	0.05	47.81 ± 4.98 a	65.08 ± 1.99 b	29.31 ± 2.68 b	39.76 ± 0.61 b
	0.025	33.17 ± 5.11 b	52.48 ± 1.59 d	26.61 ± 3.83 b	34.64 ± 2.65 c
	0.0125	29.14 ± 5.48 b	46.28 ± 2.28 c	18.64 ± 2.81 c	21.90 ± 2.21 d
Flower/fruit head	0.1	46.49 ± 6.11 a	63.04 ± 1.51 a	27.92 ± 4.54 a	39.54 ± 4.70 a
	0.05	45.31 ± 1.39 a	53.31 ± 4.70 b	26.61 ± 3.83 a	31.85 ± 3.66 b
	0.025	31.92 ± 3.10 b	44.36 ± 4.18 c	22.59 ± 4.66 ab	26.19 ± 2.24 bc
	0.0125	23.94 ± 2.06 c	34.72 ± 4.31 d	17.32 ± 2.52 b	20.99 ± 0.70 c
*F* value	Plant part	71.13 **	208.47 **	52.98 **	294.01 **
	Concentration	79.00 **	221.59 **	34.40 **	220.92 **
	Interaction	1.48 ^ns^	3.87 **	9.19 **	14.18 **

Data are expressed as mean ± standard deviation. Different letters within the same column indicate significant differences at *p* < 0.05 for each part. ^ns^ and ** indicate *p* > 0.05, and *p* ≤ 0.01, respectively.

**Table 2:** Inhibitory rate (%) of *Cirsium vulgare* extracts on the growth of *Bidens pilosa* and *Digitaria sanguinalis*.

Plant Part	Concentration (g·mL^−1^)	*Bidens pilosa*			*Digitaria sanguinalis*		
		Shoot Length	Root Length	Biomass	Shoot Length	Root Length	Biomass
Root	0.1	60.80 ± 0.22 a	48.90 ± 0.64 a	54.37 ± 1.95 a	43.74 ± 0.25 a	50.20 ± 0.86 a	43.51 ± 1.08 a
	0.05	47.39 ± 0.60 b	41.76 ± 0.78 b	36.23 ± 1.63 b	40.68 ± 0.46 b	35.25 ± 0.37 b	29.23 ± 2.64 b
	0.025	12.93 ± 0.63 c	35.77 ± 0.99 c	19.16 ± 1.18 c	23.05 ± 0.76 c	16.65 ± 0.27 c	16.89 ± 1.52 c
	0.0125	5.68 ± 0.28 d	12.01 ± 0.49 d	8.00 ± 2.00 d	4.13 ± 0.58 d	9.09 ± 0.46 d	5.84 ± 1.24 d
Stem	0.1	78.82 ± 0.25 a	72.00 ± 0.64 a	79.11 ± 1.36 a	52.95 ± 0.36 a	60.75 ± 0.53 a	68.17 ± 2.653 a
	0.05	50.85 ± 0.62 b	53.39 ± 0.47 b	63.24 ± 0.32 b	46.38 ± 0.41 b	52.83 ± 0.39 b	53.90 ± 2.35 b
	0.025	16.36 ± 0.72 c	41.47 ± 0.39 c	51.22 ± 1.04 d	28.47 ± 0.33 c	44.49 ± 0.37 c	43.51 ± 2.36 c
	0.0125	7.24 ± 0.19 d	22.03 ± 0.92 d	43.90 ± 1.21 d	8.84 ± 0.94 d	37.96 ± 0.19 d	31.17 ± 0.467 d
Leaf	0.1	82.27 ± 0.95 a	81.28 ± 0.49 a	84.67 ± 1.98 a	73.02 ± 0.47 a	75.21 ± 0.21 a	81.19 ± 2.24 a
	0.05	53.88 ± 0.37 b	58.95 ± 0.56 b	78.05 ± 1.16 b	51.99 ± 0.67 b	68.12 ± 0.34 b	70.77 ± 1.60 b
	0.025	31.34 ± 0.38 c	48.40 ± 0.81 c	63.76 ± 1.02 c	31.69 ± 0.61 c	60.48 ± 0.23 c	66.24 ± 1.80 c
	0.0125	12.55 ± 0.74 d	40.19 ± 0.92 d	55.41 ± 1.38 d	10.27 ± 0.25 d	44.20 ± 0.66 d	57.15 ± 3.120 d
Flower/fruit head	0.1	93.88 ± 0.32 a	87.79 ± 0.90 a	88.51 ± 1.22 a	75.99 ± 0.73 a	68.90 ± 0.43 a	77.28 ± 1.15 a
	0.05	73.61 ± 0.22 b	74.09 ± 0.28 b	80.15 ± 1.58 b	64.96 ± 0.71 b	65.67 ± 0.38 b	64.95 ± 0.97 b
	0.025	32.38 ± 0.37 c	49.93 ± 0.62 c	70.40 ± 2.15 c	42.03 ± 0.63 c	51.69 ± 0.52 c	51.30 ± 1.07 c
	0.0125	19.21 ± 0.98 d	42.22 ± 0.34 d	64.45 ± 1.25 d	32.14 ± 0.63 d	42.06 ± 0.37 d	49.34 ± 1.68 d
*F* value	Plant part	5099.06 **	5478.27 **	3172.93 **	5849.60 **	18575.13 **	1720.37 **
	Concentration	50371.69 **	11794.07 **	1625.24 **	21036.80 **	14604.90 **	833.36 **
	Interaction	334.32 **	254.17 **	34.57 **	295.03 **	396.99 **	13.16 **

Data are expressed as mean ± standard deviation. Different letters within the same column indicate significant differences at *p* < 0.05 for each part. ** indicates *p* ≤ 0.01.

**Table 3:** Allelopathic response index of *Bidens pilosa* due to four aqueous extracts of *Cirsium vulgare*.

Plant Part	Concentration (g·mL^−1^)	Germination Rate	Germination Index	Shoot Length	Root Length	Biomass	Synthetical Allelopathic Index
Root	0.1	−0.266 ± 0.033 b	−0.460 ± 0.033 c	−0.608 ± 0.002 d	−0.489 ± 0.006 d	−0.544 ± 0.019 d	−0.473 ± 0.008 d
	0.05	−0.212 ± 0.035 b	−0.313 ± 0.036 b	−0.474 ± 0.006 c	−0.418 ± 0.008 c	−0.362 ± 0.016 c	−0.356 ± 0.015 c
	0.025	−0.159 ± 0.037 ab	−0.162 ± 0.042 a	−0.129 ± 0.006 b	−0.358 ± 0.010 b	−0.192 ± 0.012 b	−0.200 ± 0.018 b
	0.0125	−0.090 ± 0.085 a	−0.095 ± 0.055 a	−0.057 ± 0.003 a	−0.120 ± 0.005 a	−0.080 ± 0.020 a	−0.088 ± 0.030 a
Stem	0.1	−0.439 ± 0.036 c	−0.607 ± 0.013 d	−0.788 ± 0.002 d	−0.720 ± 0.006 d	−0.791 ± 0.013 d	−0.669 ± 0.011 d
	0.05	−0.332 ± 0.039 bc	−0.486 ± 0.080 c	−0.509 ± 0.006 c	−0.534 ± 0.005 c	−0.632 ± 0.003 c	−0.499 ± 0.026 c
	0.025	−0.226 ± 0.043 ab	−0.316 ± 0.028 b	−0.164 ± 0.007 b	−0.415 ± 0.004 b	−0.512 ± 0.010 b	−0.326 ± 0.012 b
	0.0125	−0.144 ± 0.082 a	−0.198 ± 0.042 a	−0.072 ± 0.002 a	−0.220 ± 0.009 a	−0.439 ± 0.012 a	−0.215 ± 0.023 a
Leaf	0.1	−0.533 ± 0.025 b	−0.695 ± 0.005 d	−0.823 ± 0.009 d	−0.813 ± 0.005 d	−0.847 ± 0.020 d	−0.742 ± 0.003 d
	0.05	−0.478 ± 0.050 b	−0.651 ± 0.020 c	−0.539 ± 0.004 c	−0.589 ± 0.006 c	−0.781 ± 0.012 c	−0.608 ± 0.012 c
	0.025	−0.332 ± 0.051 a	−0.525 ± 0.016 b	−0.313 ± 0.004 b	−0.484 ± 0.008 b	−0.638 ± 0.010 b	−0.458 ± 0.012 b
	0.0125	−0.291 ± 0.055 a	−0.463 ± 0.023 a	−0.126 ± 0.007 a	−0.402 ± 0.009 a	−0.554 ± 0.014 a	−0.367 ± 0.013 a
Flower/fruit head	0.1	−0.465 ± 0.061 c	−0.630 ± 0.015 d	−0.939 ± 0.003 d	−0.878 ± 0.010 d	−0.885 ± 0.012 d	−0.759 ± 0.014 d
	0.05	−0.453 ± 0.014 c	−0.533 ± 0.047 c	−0.736 ± 0.002 c	−0.741 ± 0.003 c	−0.801 ± 0.016 c	−0.653 ± 0.012 c
	0.025	−0.319 ± 0.031 b	−0.444 ± 0.042 b	−0.324 ± 0.004 b	−0.499 ± 0.006 b	−0.704 ± 0.021 b	−0.458 ± 0.016 b
	0.0125	−0.239 ± 0.021 a	−0.347 ± 0.043 a	−0.192 ± 0.010 a	−0.422 ± 0.003 a	−0.644 ± 0.012 a	−0.369 ± 0.013 a

Data are expressed as mean ± standard deviation. Different letters within the same column indicate significant differences at *p* < 0.05 for each plant part.

**Table 4:** Allelopathic response index of *Digitaria sanguinalis* due to four aqueous extracts of *Cirsium vulgare*.

Plant Part	Concentration (g·mL^−1^)	Germination Rate	Germination Index	Shoot Length	Root Length	Biomass	Synthetical Allelopathic Index
Root	0.1	−0.186 ± 0.050 a	−0.207 ± 0.021 c	−0.437 ± 0.002 d	−0.502 ± 0.009 d	−0.435 ± 0.011 d	−0.342 ± 0.022 d
	0.05	−0.145 ± 0.065 a	−0.170 ± 0.018 c	−0.407 ± 0.005 c	−0.353 ± 0.004 c	−0.292 ± 0.026 c	−0.273 ± 0.015 c
	0.025	−0.133 ± 0.029 a	−0.098 ± 0.027 b	−0.230 ± 0.008 b	−0.166 ± 0.003 b	−0.169 ± 0.015 b	−0.159 ± 0.012 b
	0.0125	−0.119 ± 0.049 a	−0.036 ± 0.039 a	−0.041 ± 0.006 a	−0.091 ± 0.005 a	−0.058 ± 0.012 a	−0.069 ± 0.017 a
Stem	0.1	−0.213 ± 0.040 a	−0.323 ± 0.019 c	−0.529 ± 0.004 d	−0.607 ± 0.005 d	−0.682 ± 0.027 d	−0.471 ± 0.010 d
	0.05	−0.200 ± 0.048 a	−0.263 ± 0.028 b	−0.464 ± 0.004 c	−0.528 ± 0.004 c	−0.539 ± 0.024 c	−0.399 ± 0.011 c
	0.025	−0.186 ± 0.050 a	−0.209 ± 0.027 a	−0.285 ± 0.003 b	−0.445 ± 0.004 b	−0.435 ± 0.024 b	−0.312 ± 0.014 b
	0.0125	−0.160 ± 0.004 a	−0.161 ± 0.016 a	−0.088 ± 0.009 a	−0.380 ± 0.002 a	−0.312 ± 0.005 a	−0.220 ± 0.002 a
Leaf	0.1	−0.533 ± 0.033 c	−0.620 ± 0.020 d	−0.730 ± 0.005 d	−0.752 ± 0.002 d	−0.812 ± 0.022 c	−0.689 ± 0.008 d
	0.05	−0.293 ± 0.027 b	−0.398 ± 0.006 c	−0.520 ± 0.007 c	−0.681 ± 0.003 c	−0.708 ± 0.016 b	−0.520 ± 0.009 c
	0.025	−0.266 ± 0.038 b	−0.346 ± 0.027 b	−0.317 ± 0.006 b	−0.605 ± 0.002 b	−0.662 ± 0.018 b	−0.439 ± 0.015 b
	0.0125	−0.186 ± 0.028 a	−0.219 ± 0.022 a	−0.103 ± 0.002 a	−0.442 ± 0.007 a	−0.572 ± 0.031 a	−0.304 ± 0.012 a
Flower/fruit head	0.1	−0.279 ± 0.045 b	−0.395 ± 0.047 c	−0.760 ± 0.007 d	−0.689 ± 0.004 d	−0.773 ± 0.011 c	−0.579 ± 0.021 d
	0.05	−0.266 ± 0.038 b	−0.318 ± 0.037 b	−0.650 ± 0.007 c	−0.657 ± 0.004 c	−0.649 ± 0.010 b	−0.508 ± 0.014 c
	0.025	−0.226 ± 0.047 ab	−0.262 ± 0.022 ab	−0.420 ± 0.006 b	−0.517 ± 0.005 b	−0.513 ± 0.011 a	−0.388 ± 0.013 b
	0.0125	−0.173 ± 0.025 a	−0.210 ± 0.007 a	−0.321 ± 0.006 a	−0.421 ± 0.004 a	−0.493 ± 0.017 a	−0.324 ± 0.009 a

Data are expressed as mean ± standard deviation. Different letters within the same column indicate significant differences at *p* < 0.05 for each plant part.

**Table 5:** Main phenolic acid and flavonoid compounds of methanol extracts of *Cirsium vulgare*.

No.	Compound	Retention Time (min)	Percentage (%)	Molecular Ion (*m*/*z*)	Class I
1	3-(4-hydroxy-3-methoxyphenyl)prop-2-enoyl 1,3,4,5-tetrahydroxycyclohexane-1-carboxylate	3.4	2.17	368.1	Phenolic acid
2	2-[3-[3-(3,4-dihydroxyphenyl)prop-2-enoyloxy]-4-hydroxy-5-oxooxolan-2-yl]-2-hydroxyacetic acid	2.4	2.16	354.1	Phenolic acid
3	Cyanidin 3-O-(6-O-p-coumaroyl) glucoside	3.8	2.04	595.1	Flavonoid
4	Kaempferol-3-O-glucorhamnoside	3.9	2.02	594.2	Flavonoid
5	Luteolin-7-O-rutinoside	3.8	1.87	594.2	Flavonoid
6	Chlorogenate	2.9	1.84	354.1	Phenolic acid
7	Poriolin	4.0	1.48	448.1	Flavonoid
8	3-O-p-Coumaroylquinic acid	3.1	1.26	338.1	Phenolic acid
9	6-Hydroxyluteolin 7-(6″-malonylglucoside)	3.9	1.18	550.1	Flavonoid
10	6-Hydroxylignan-6-O-(6″-O-malonyl) glucoside	3.9	1.17	550.1	Flavonoid
11	4′-Hydroxy-7-methoxyflavanone-6-O-glucoside	3.9	1.12	448.1	Flavonoid
12	Luteolin-4′-O-glucoside	4.1	1.10	448.1	Flavonoid
13	Hesperetin-3′-O-glucoside	3.9	1.09	464.1	Flavonoid
14	Rhodionidin	3.6	1.08	610.2	Flavonoid
15	Scutellarein 6-glucoside	4.0	1.05	448.1	Flavonoid
16	Kaempferol 3-O-(2″-O-malonyl) glucoside	4.3	1.05	534.1	Flavonoid
17	Kaempferol-3-O-neohesperidoside	3.8	1.04	594.2	Flavonoid
18	Galangin-7-O-glucuronic acid	4.1	1.03	446.1	Flavonoid
19	Rutin trihydrate	3.7	1.02	664.2	Flavonoid
20	Kaempferol-4′-O-glucoside	4.1	1.01	448.1	Flavonoid
21	Kaempferol 3-O-(2″-rhamnosyl)glucuronide	3.6	1.00	608.1	Flavonoid
22	Apigenin 7-O-neohesperidoside	4.0	0.96	578.2	Flavonoid
23	Kaempferol-3-O-galactoside	4.1	0.95	448.1	Flavonoid
24	Kaempferol 3-O-glucoside	4.1	0.94	448.1	Flavonoid
25	Herbacetin 4′-glucoside	3.6	0.94	464.1	Flavonoid
26	Isoquercitrin	3.8	0.92	464.1	Flavonoid
27	Luteolin 7-O-beta-D-glucoside	4.1	0.92	448.1	Flavonoid
28	Quercetin-3-O-robinobioside	3.6	0.92	610.2	Flavonoid
29	Baicalin	4.1	0.92	446.1	Flavonoid
30	Apigenin-7-O-glucuronide	4.1	0.92	446.1	Flavonoid

## Data Availability

The original contributions presented in this study are included in the article. Further inquiries can be directed to the corresponding author(s).
